# Easy Access to a Cyclic Key Intermediate for the Synthesis of Trisporic Acids and Related Compounds

**DOI:** 10.3390/molecules19021748

**Published:** 2014-02-03

**Authors:** José A. González-Delgado, Gustavo Escobar, Jesús F. Arteaga, Alejandro F. Barrero

**Affiliations:** 1 Department of Organic Chemistry and Institute of Biotechnology, University of Granada, Avda Fuentenueva s/n, 18071 Granada, Spain; 2 CIQSO–Center for Research in Sustainable Chemistry and Department of Chemical Engineering, Physical Chemistry and Organic Chemistry, University of Huelva, Avda 3 Marzo s/n, 21071 Huelva, Spain; 3 University of Antioquia, Calle 70 N° 52-21, Medellín, Colombia

**Keywords:** trisporoids, apocarotenoid, domino reaction, stereoselective synthesis

## Abstract

The synthesis of a cyclohexane skeleton possessing different oxygenated functional groups at C–3, C–8 and C–9, and a Δ^1,6^-double bond has been accomplished in 10 steps with an overall 17% yield. This compound is a key intermediate for access to a wide range of compounds of the bioactive trisporoid family. The synthetic sequence consists of the preparation of a properly functionalized epoxygeraniol derivative, and its subsequent stereoselective cyclization mediated by Ti(III). This last step implies a domino process that starts with a homolytic epoxide opening followed by a radical cyclization and regioselective elimination. This concerted process gives access to the cyclohexane moiety with stereochemical control of five of its six carbon atoms.

## 1. Introduction

**Figure 1:**
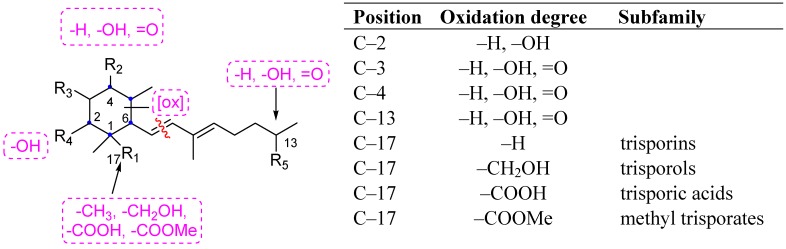
Structural diversity in the family of trisporoids.

Trisporic acids (Figure 1), their precursors and derivatives are an interesting group of bioactive natural products biosynthetically derived from the degradative oxidation of β-carotene. They act as inducers of carotenogenesis as well as pheromones involved in the regulation of different stages of sexual development and stimulation of the production of zygophores in the fungal phylum Zygomycota and other Mucorales. These facts are well reported for different species of fungi such as *Blakeslea trispora* (Choaneforaceae family) [1,2,3], *Phycomyces blakesleeanus* (Phycomycetaceae family) [4], *Mucor mucedo* (Mocuraceae family) [2,5,6,7], and the homothallic *Zygorhynchus moelleri* (Mocuraceae family) [8]. Furthermore several species of Mortierella (Mortierallale order) produce trisporoids that can induce sexual responses in *Mucor mucedo* and *Phycomyces blakesleeanus* [9]. Recently it has been demonstrated that the recognition between the parasite fungi *Parasitella parasitica* (Mocuraceae family) and the host *Absidia glauca* is mediated by trisporoids which are responsible for sexual phenomena [10,11]. Furthermore, evidence for the existence of genes of trisporoids synthesis in Glomus-like fungi (*Phylum Glomeromycota*) has been reported [12]. In recent years, a number of studies for implementing assays for the isolation and characterization of trisporoids have been developed [13,14,15,16] because they occur in fungi in very low concentrations and are chemically characterized for their relative instability. Sometimes this characteristic feature of these hormones hinders their direct structural characterization. Hence, the availability of this important class of compounds through chemical synthesis is a pre-requirement for their correct structural characterization and for carrying out physiological studies.

**Scheme 1:**
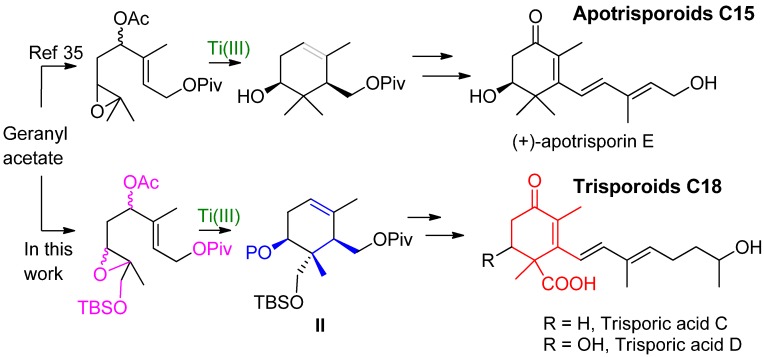
Ti(III)-mediated synthesis of apotrisporoids and trisporoids based on bioinspired cyclizations.

Several routes to trisporoids such as trisporins, methyl trisporates, trisporols, and free trisporic acids have been developed and reported [17,18,19,20,21,22,23,24,25,26,27,28,29,30,31,32,33,34]. The disadvantage of most routes is the large number of steps required for the synthesis of the final targets and the lack of flexibility to produce early and late trisporoids along the same protocol using common intermediates. The approaches by Boland [31] and Rodríguez-García [32] are examples of modern strategies that carry out the synthesis of compounds of this family by using organometallic couplings. However, they lead only to early trisporoids without oxygen functions in the gem-dimethyl group of the cyclohexane. The first total synthesis of a number of apotrisporoids C15 (monocyclofarnesoids) has been accomplished for the first time in our laboratories [35], enabling the corroboration of the relative stereochemistry and the assignation of the absolute configuration for these compounds (Scheme 1). This approach is based on a bio-inspired cyclization constituted by a domino process: homolytic oxyrane opening, radical cyclization, alkyltitanium formation and final regioslective elimination and a HWE olefination to attach the dienic side chain as key steps.

**Scheme 2:**
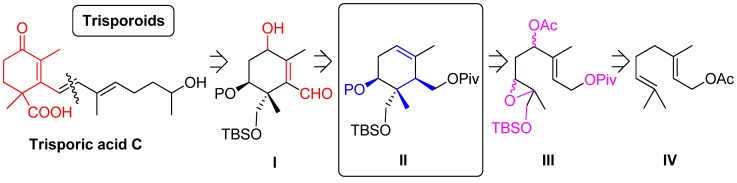
Retrosynthetic approach.

Following this strategy, herein a significant advance in the development of a selective synthesis of highly functionalized key intermediates such as **II** (Scheme 1 and Scheme 2) is described. These may be considered as effective precursors for the synthesis of trisporic acids and their corresponding alcohol derivatives (trisporols), mainly for their use as a standard either in subsequent identifications, quantifications, and biological activity tests (as fungi pheromones). Its selective preparation supposes an extension and generalization of the above outlined previous approach. For trisporic acids or trisporols the methodology proposed constitutes a new synthetic route and involves the access to key intermediate type **II** starting from commercial geranyl acetate.

Having in consideration the objective to achieve straightforward functional modifications at C–2, C–4, C–5, C–13 and C–17 (Figure 1), such approach would therefore open the door to the synthesis of the majority of the known natural trisporoids C18; not only to early trisporoids such as trisporins but also to late ones such as trisporols, trisporic acid and methyl trisporates.

## 2. Results and Discussion

The retrosynthetic approach towards natural trisporic acids and trisporols (as trisporic acid C in Scheme 2) is based on a disconnection through a synthon of ten carbon atoms whose cyclohexane structure will be provided by the intermediate **I** (Scheme 2). **I** could be formed from intermediate **II** by using well known transformations such as epoxidation of the trisubstituted double bond, opening of the oxirane in basic medium, selective deprotection and subsequent oxidation. **II** derives from a Ti(III)-mediated radical cyclization of the acyclic precursor **III**. Finally the highly functionalized acyclic epoxide **III** could be obtained from commercial geranyl acetate (**IV**).

**Scheme 3:**
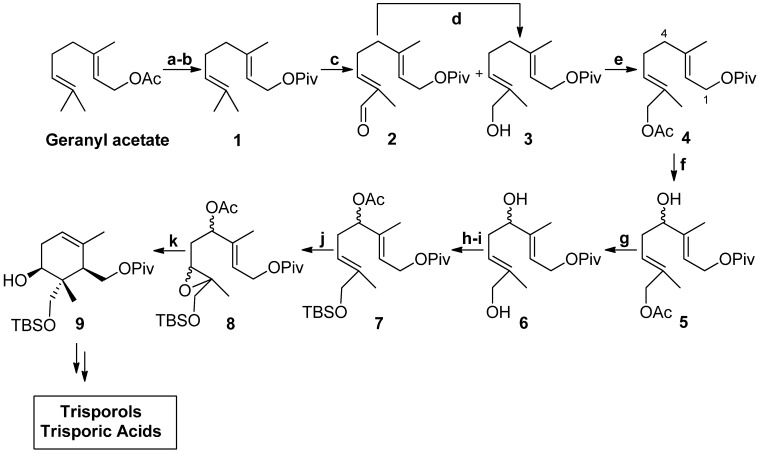
Synthesis of polyfunctionalized cyclohexane **9**, key structure towards trisporic acids family.

To achieve the access towards intermediate **II**, a synthetic sequence of ten steps (Scheme 3) was realized, in which the key step is a stereoselective cyclization mediated by Cp_2_Ti^III^Cl [36] by means of the homolytic opening of the highly functionalized epoxypolyprene (**8**, **III** in Scheme 2) [37,38,39,40]. In this regard the preparation of **8 **(or **III**) in good yield constitutes a useful synthetic novelty which could facilitate the synthesis of either trisporoids or labdanes. Globally the synthetic sequence presents two different regioselective allylic oxidations that can also be considered as critical steps in the entire route. In the first one, we decided to use catalytic SeO_2_ [41] bearing in mind that the –CH_3_ at the end of the chain is the object of the reaction, a process well known to occur properly. In the second one, which aims to functionalize a –CH_2_ placed inside the carbon chain, the reaction occurs slowly and is more difficult to process with respect to the above oxidation reaction. In this case excess SeO_2_ together with aqueous EtOH has been used [42,43,44].

*Reagents and conditions*: a. K_2_CO_3_, MeOH, 25 °C, 10 min, 99%; b. PivCl, Imidazole, Pyridine, rt, 1 h, 99%; c. SeO_2_, *t*-BuOOH, DCM, 0 °C, 3 h, 84% based on recovered starting material; d. NaBH_4_, MeOH, rt, 30 min, 95%; e. Ac_2_O, Pyridine, DMAP, rt, 3 h, 85%; f. SeO_2_, aq. EtOH, reflux, 2 h, 72% based on recovered starting material, g. K_2_CO_3_, MeOH, 0 °C, 2 h, 99%; h. TBSCl, Imidazole, DMF, rt, 3 h, 79%; i. Ac_2_O, Pyridine, DMAP, rt, 1 h, 73%; j. *m*-CPBA, DCM, 0 °C, 2 h 30 min, 90%; k. Cp_2_TiCl_2_, Mn, THF, 40 °C, 6 h, 70%.

Sequentially, the first stage involves the protective group exchange of the starting material to arrive at compound **1** (98% yield). Allylic oxidation of **1** with catalytic amounts of SeO_2_ in the presence of equimolar *t*-BuOOH led to a mixture of the hydroxyester **3** together with the over-oxidation product **2**. This aldehyde **2** can be reduced to the corresponding alcohol in an efficient manner by treatment with NaBH_4_ in MeOH. The final yield of alcohol **3** obtained by this oxidation protocol is 84%, taking into account the recycling of the unreacted starting material. Subsequent acetylation of the hydroxyester **3** with Ac_2_O in pyridine led to acetate **4** with 85% yield. Next we attempted to oxidize the –CH_2_ placed inside the carbon chain (at C–4) of **4** by means of excess SeO_2_ in aqueous EtOH. Different experiments modifying either the ratio of H_2_O–EtOH and/or the temperature were performed to increase the yield of the reaction (Table 1). The best results were obtained when using 97.5% EtOH in water yielding 72% of the secondary alcohol (**5**); taking into account the recycled unreacted starting material. Compound **5** is selectively deacetylated to give the diol **6** (99% yield) by using K_2_CO_3_ and MeOH.

**Table 1:** Optimization of the conditions of allylic oxidation of **4**.

Entry	EtOH purity [%]	Temperature [°C]	Time [min]	Ratio [5:4]	Yield [%]
1	40	Reflux	360	1.0:10	5
2	65	Reflux	360	1.0:7.0	7
3	80	Reflux	360	1.0:4.5	15
4	90	Reflux	120	1.0:4.0	36
5	90	60	240	1.0:3.2	24
6	99.8	Reflux	100	1.0:1.7	51 *^a^*
7	95.0	60	35	1.0:1.0	49 *^a^*
8	97.5	60	120	1.0:1.0	67 *^a^*
9	97.5	Reflux	120	1.1:1.0	72 *^a^*

*^a^* Yield based on recovering the starting material **4**.

At this point, we tested the selective protection of the primary alcohol of **6** by means of TBDMSCl. This reagent should react chemoselectively due to steric constraints in the analogous process with secondary alcohols. However, to avoid over-protection an equimolar proportion of TBDMSCl with respect to the starting material was used. This, together with the strict temperature control (0 °C throughout the process), enabled the preparation of the desired hydroxy-silyl derivative in 89% yield. The secondary –OH was then acetylated to obtain **7** in a yield of 73%. The selective location of the protective groups in different segments of the molecule promotes the electronic differentiation of the monoterpenic derivative and this is of primordial importance for the subsequent epoxidation reaction. An electron donating protective group (a silyl ether) has been located near the Δ^6,7^ double bond, while electron withdrawing protective groups (such as pivaloyl and acetate) have been placed around the Δ^2,3^ double bond. The latter, moreover, has been specifically selected to act as a potential leaving group when carrying out the Ti(III)-mediated radical cyclization of the acyclic precursor, which is the last step of the herein performed synthetic sequence.

**Scheme 4:**
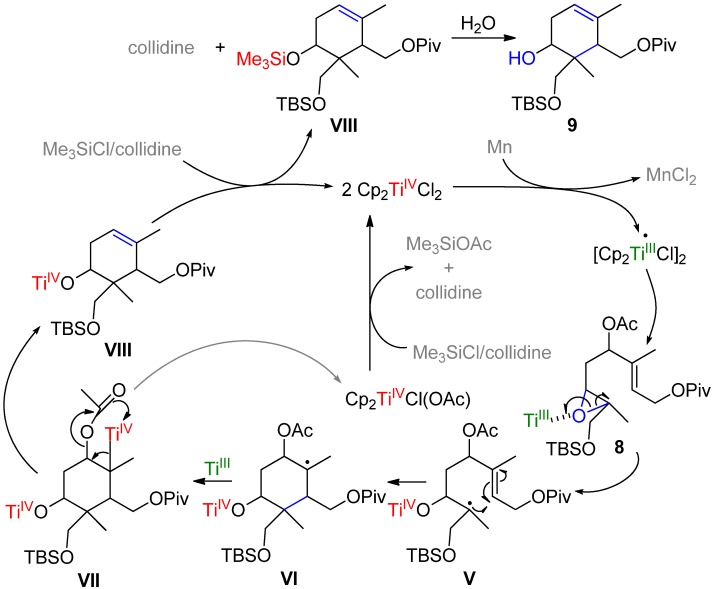
Mechanistic proposal for Ti(III)-mediated catalytic domino process towards **9**: homolytic epoxide opening of **8**, radical cyclization and selective elimination.

The selectively activated Δ^6,7^ double bond was epoxidized by means of *m*-CPBA to obtain compound **8** with 90% yield. Finally, the acyclic epoxide **8**, which also corresponds to the intermediate **III** of Scheme 1, was cyclized following the Ti(III)-mediated methodology [39,45,46,47,48,49,50]. In this case catalytic conditions [51] were employed, involving 0.3 equivalents of Ti(III) and 8.0 equivalents of Mn in THF (0.1 M), with 2,4,6–collidine/TMSCl as regenerating system. To promote cyclization process, the mixture was further heated to 40 °C. The catalytic cyclization reaction can be settled through the intermediates described in Scheme 3. The transformation begins with the formation of a Lewis acid-base complex between the oxirane group of **8** and Cp_2_TiCl [37,52], the reagent equivalent of Ti(III). This complex promotes the homolytic opening of the epoxide via one-electron-transfer from the Ti(III) atom, resulting in the formation of the carbonated acyclic β-titanoxy-radical **V**. **V** evolves by cyclizing via radical attack at the Δ^2,3^ double bond, through a 6-*endo*-trig process favored by the electronic structure of the tri-substituted double bond, leading to the more substituted radical **VI**. This radical reacts with another Ti(III) forming the alkyl-Ti(IV) species **VII**, which through elimination of Cp_2_TiCl(OAc) leads with complete regioselectivity to the compound possessing the tri-substituted double bond Δ^5,6^ (**VIII**, Scheme 4). Cp_2_TiCl_2_ can be regenerated by means of the 2,4,6–collidine/TMSCl system either from the Cp_2_Ti(IV)Cl(OAc) generated in the –OAc elimination step or from the titanium alkoxide of **VIII**.

**Scheme 5:**
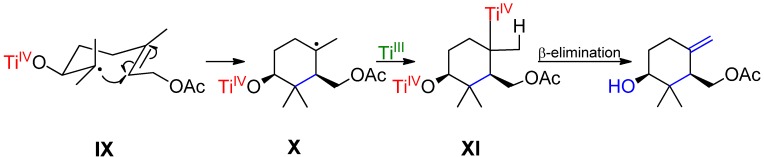
Usual cyclization pathway in non-functionalized at C–4 derivatives leading to exocyclic double bonds.

It should be pointed out that the Ti(III)-mediated radical cyclization reactions employing substrates without secondary acetate groups at C–4 lead regioselectively to exocyclic double bonds (Scheme 5) [45,47,48,49,50]. In contrast, in this study it has been proven that it is possible to change selectively the direction of the formation of the olefin by using a suitable leaving group having in mind the affinity for the oxygen of the Ti atom.

The structure and stereochemistry of the cyclization product (**9**) is easily deduced from the spectroscopic data, in particular NMR (Table 2).

**Table 2:** ^1^H-NMR and ^13^C-NMR data for **9**.

Position[NOE]	^1^H				^13^C	Representative NOE effects found in 9.
	δ [ppm]	signal		*J* [Hz]	δ [ppm]	
**1**	5.39	1H	bs		121.1	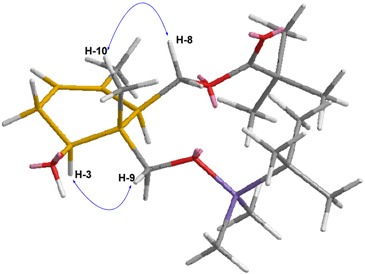
**2**	2.22	2H	m		30.8	
**3**α	3.85	1H	dd	8.4 5.6	71.5	
**4**					41.4	
**5**	2.02	1H	m		43.1	
**6**					132.6	
**7**	1.68	3H			22.0	
**8**β	4.07	1H	dd	11.8 4.4	62.7	
	4.30	1H	dd	11.8 4.3		
**9**α	3.53 3.68	1H 1H	d d	10.0 10.0	68.9	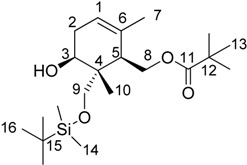
**10**β	0.88	1H	s		12.0	
**11**					178.5	
**12**					38.8	
**13**	1.19	9H	s		27.2	
**14**	0.07 0.06	3H 3H	s s		–5.5	
**15**	5.39	1H	bs		18.2	
**16**	2.22	2H	m		25.9	

The relative configuration is determined by means of NOE experiments. NOE effects can be observed between the protons H–8 and H–10 as well as between the protons H–9 and H–3. This allows us determining that each pair of H is placed in a *syn* disposition. Furthermore, considering the proposed reaction mechanism, these evidences defined the stereochemistry of the various stereogenic centers of **9** (Table 2). In this way, it becomes clear that this cyclization process occurs with an excellent regioselectivity control, both in the ring closure and in the formation of the tri-substituted double bond. A good stereoselectivity in the formation of three stereogenic centers (located consecutively in the cyclohexane structure of **9**) is obtained. This is consistent with the precedents in this kind of cyclizations [53].

Regarding the consequences of this approach for investigations at the biological level, the development of a short synthetic process towards the intermediate **9** will allow to access different compounds of both families of trisporoidss C18 and monocyclofarnesyl derivatives C15 (or apotrisporoids). Thus, the hydroxyl located at C–3 will provide access to trisporoids of the D series, or by means of a deoxygenation to those of C, B or A series. Furthermore the Δ^1,6^ double bond enables the integration of either a carbonyl or hydroxyl group at C–6, as present in the trisporic acids and dihydrotrisporols, respectively. The availability of these molecules will facilitate the testing of their biological activity. These studies will help to find the true sex pheromones of both (+) and (–) genus in these fungi, and also the specific molecules responsible of carotenogenesis.

## 3. Experimental

### 3.1. General

All NMR spectra (δ values, ppm) were recorded with Varian Direct-Drive 600 (^1^H 600 MHz/^13^C 150 MHz), Varian Direct-Drive 500 (^1^H 500 MHz/^13^C 125 MHz), Varian Direct-Drive 400 (^1^H 400 MHz/^13^C 100 MHz) and Varian Inova Unity 300 (^1^H 300 MHz/^13^C 75 MHz) spectrometers. Tetramethylsilane (TMS) was used as an external reference for recording ^1^H (of residual proton; δ = 7.26 ppm) and ^13^C (δ = 77.0 ppm) spectra in CDCl_3_. Chemical shift multiplicities are reported as s = singlet, d = doublet, t = triplet, q = quartet, m = multiplet and br = broad. The accurate mass determination was carried out with a mass spectrometer equipped with a TOF, system Triwave^®^ WATERS model SYNAPG2 and an AutoSpec-Q mass spectrometer arranged in an EBE geometry (Micromass Instrument, Manchester, UK) and equipped with a FAB (LSIMS) source. The instrument was operated at 8 KV of accelerating voltage and Cs^+^ were used as primary ions. Optical rotations were measured on a Perkin-Elmer 141 polarimeter, using CH_2_Cl_2_ as the solvent. Reactions were monitored by thin layer chromatography (TLC) carried out on 0.25 mmE. Merck silica gel plates (60F-254) using UV light as the visualizing agent and a solution of phosphomolybdic acid in ethanol and heat as developing agent. Silica gel SDS 60 (35–70 mm) was used for flash column chromatography. HPLC with UV and RI detection was used. Semi-preparative HPLC separations were carried out on a column (5 µm Silica, 10 × 250 mm) at a flow rate of 2.0 mL/min in an Agilent Series 1100 instrument. All air- and water-sensitive reactions were performed in flaks flame-dried under a positive flow of argon and conducted under an argon atmosphere. The solvents used were purified according to standard literature techniques and stored under argon. THF and toluene were freshly distilled immediately prior to use from sodium/benzophenone and strictly deoxygenated for 30 min under argon for each of the Cp_2_TiCl_2_/Mn or Zn reactions. Reagents were purchased at the higher commercial quality and used without further purification, unless otherwise stated.

### 3.2. Experimental Procedures

*Synthesis of* (*E*)-*3,7-dimethylocta-2,6-dien-1-yl pivalate* (**1**). To a stirred solution of geraniol (7.0 g, 45.75 mmol) in pyridine (26 mL), imidazole (0.622 g, 9.15 mmol) and PivCl (7.3 mL, 59.48 mmol) were added at room temperature. After stirring for 1 h (TLC monitoring), the mixture was diluted with CH_2_Cl_2_ and water and extracted with CH_2_Cl_2_. The combined organic layer was washed with 2 N HCl, brine, dried over anhydrous Na_2_SO_4_ and concentrated under reduced pressure. The resulting crude was purified by flash chromatography (hexane/*t*-BuOMe, 1:1) on silica gel to afford **1** (10.8 g, 45.29 mmol, 99% yield). ^1^H-NMR (400 MHz, CDCl_3_): δ 5.32 (1H, t), 5.08 (1H, t), 4.56 (2H, d), 2.08 (2H, q), 2.04 (2H, t), 1.69 (3H, s), 1.67 (3H, s), 1.60 (3H, s) and 1.18 (9H, s) ppm.

*Synthesis of (2E,6E)-8-hydroxy-3,7-dimethylocta-2,6-dien-1-yl pivalate* (**3**). A mixture of SeO_2_ (1,012 mg, 9.12 mmol), *tert*-butylhydroperoxide 5.0–6.0 M in decane (3.4 mL, 18.52 mmol) and DCM (49 mL) was stirred at 0 °C for 20 min. Then, **1** was added (6.588 g, 27.64 mmol). The mixture was stirred for 3 h, diluted with 25 mL of DCM, washed with water for three times and brine, dried over anhydrous Na_2_SO_4_ and concentrated under reduced pressure. The resulting crude was purified by flash chromatography (hexane/*t*-BuOMe, 2:1) on silica gel to afford **3** (5.905 g, 23.22 mmol, 84% yield after recovering starting material). ^1^H-NMR (400 MHz, CDCl_3_): δ 5.36 (1H, t), 5.34 (1H, t), 4.56 (2H, d), 3.98 (2H, s), 2.16 (2H, q), 2.08 (2H, t), 1.70 (3H, s), 1.68 (3H, s) and 1.12 (9H, s) ppm. HRFABMS: calcd for C_15_H_26_O_3_Na [M+Na]^+^ 277.1780, found: 277.1769.

*Synthesis of (2E,6E)-8-acetoxy-3,7-dimethylocta-2,6-dien-1-yl pivalate* (**4**). Acetic anhydride (2.34 ml, 24.79 mmol) and 4-dimethylaminopyridine (5.0 mg) were added to solution of **3** (3.153 g, 12.4 mmol) in pyridine (33 mL). The mixture was kept at room temperature for 3 h, diluted with 50 mL of ice-water, extracted with *t*-BuOMe and washed with 1 N HCl for three times and brine. Consequently, the organic layer was dried over anhydrous Na_2_SO_4_ and concentrated under reduced pressure. The resulting crude was purified by flash chromatography (hexane/*t*-BuOMe, 4:1) on silica gel to afford **4** (3.124 g, 10.54 mmol, 85% yield). ^1^H-NMR (400 MHz, CDCl_3_): δ 5.38 (1H, t), 5.26 (1H, t), 4.50 (2H, d), 4.38 (2H, s), 2.10 (2H, t), 2.05 (m, 2H), 2.00 (3H, s), 1.63 (3H, s), 1.59 (3H, s) and 1.12 (9H, s) ppm. ^13^C-NMR (100 MHz, CDCl_3_): δ 178.5, 170.9, 141.0, 130.4, 128.8, 119.1, 70.1, 61.2, 38.8, 38.7, 27.2, 25.9, 20.9, 16.4 and 13.9 ppm. HRFABMS: calcd for C_17_H_28_O_4_Na [M+Na]^+^ 319.1885, found: 319.1890.

*Synthesis of (2E,6E)-8-acetoxy-4-hydroxy-3,7-dimethylocta-2,6-dien-1-yl pivalate* (**5**). A solution of **4** (3.122 g, 10.53 mmol) in DCM (49 mL) was added EtOH 97.5% (1.035 mL) and SeO_2_ (4.675 g, 42.14 mmol) under Ar atmosphere and refluxing. The mixture was stirred for 2 h, diluted with EtOAc, washed with brine for three times and dried over anhydrous Na_2_SO_4_. Finally, the organic phase was concentrated under reduced pressure and the resulting crude was purified by flash chromatography (hexane/*t*-BuOMe, 2:1) on silica gel to afford **5** (2.37 g, 7.58 mmol, 72% yield after recovering starting material). ^1^H-NMR (400 MHz, CDCl_3_): δ 5.52 (1H, t), 5.39 (1H, t), 4.55 (2H, d), 4.40 (2H, s), 4.02 (1H, t), 2.26 (2H, t), 2.00 (3H, s), 1.65 (3H, s), 1.62 (3H, s) and 1.12 (9H, s) ppm. ^13^C-NMR (100 MHz, CDCl_3_): δ 178.5, 170.9, 142.0, 133.0, 124.7, 120.2, 75.9, 69.9, 60.9, 49.4, 38.7, 33.6, 27.2, 27.0, 21.0, 14.2 and 12.3 ppm. HRFABMS: calcd for C_17_H_28_O_5_Na [M+Na]^+^ 335.1834, found: 335.1824.

*Synthesis of (2E,6E)-4,8-dihydroxy-3,7-dimethylocta-2,6-dien-1-yl pivalate* (**6**). To a stirred solution of **5 **(1.198 g, 3.83 mmol) in MeOH (27 mL), K_2_CO_3_ (529 mg, 3.83 mmol) was added at 0 °C. After stirring for 2 h (TLC monitoring), the mixture was diluted with brine and extracted with EtOAc. The combined organic layer was dried over anhydrous Na_2_SO_4_ and concentrated under reduced pressure. The resulting crude was purified by flash chromatography (hexane/*t*-BuOMe, 1:1) on silica gel to afford **6** (1.025 g, 3.79 mmol, 99% yield). ^1^H-NMR (400 MHz, CDCl_3_): δ 5.52 (1H, t), 5.38 (1H, t), 4.58 (2H, d), 4.04 (1H, t), 3.94 (2H, s), 2.52 (2H, s), 1.68 (3H, s), 1.62 (3H, s) and 1.18 (9H, s) ppm. ^13^C-NMR (100 MHz, CDCl_3_): δ 178.7, 142.0, 137.8, 121.0, 120.1, 76.1, 68.5, 61.1, 38.7, 33.3, 27.2 (× 3), 14.0 and 12.4 ppm. HRFABMS: calcd for C_15_H_26_O_4_Na [M+Na]^+^ 293.1729, found: 293.1738.

*Synthesis of* *(2E,6E)-4-acetoxy-8-((tert-butyldimethylsilyl)oxy)-3,7-dimethylocta-2,6-dien-1-yl pivalate* (**7**). To a stirred solution of **6** (839.0 mg, 3.1 mmol) in DMF (33 mL), imidazole (316.6 mg, 4.65 mmol) and TBSCl (561.3 mg, 3.72 mmol) were added at room temperature. After stirring for 3 h (TLC monitoring), the mixture was diluted with *t*-BuOMe and water and extracted with *t*-BuOMe. The combined organic layer was washed with 2 N HCl, brine, dried over anhydrous Na_2_SO_4_ and concentrated under reduced pressure. The resulting crude was purified by flash chromatography (hexane/*t*-BuOMe, 1:1) on silica gel to afford **7** (1.045 g, 2.45 mmol, 79% yiedl). ^1^H-NMR (400 MHz, CDCl_3_): δ 5.51 (1H, t), 5.32 (1H, t), 4.55 (2H, d), 4.02 (1H, t), 3.94 (2H, s), 2.25 (2H, t), 1.72 (1H, s), 1.64 (3H, s), 1.55 (3H, s), 1.12 (9H, s), 0.85 (9H, s) and 0.00 (6H, s) ppm. ^13^C-NMR (100 MHz, CDCl_3_): δ 178.5, 170.2, 138.2, 137.4, 122.0, 118.5, 77.7, 68.2, 60.8, 38.8, 31.1, 27.2 (× 3), 26.0 (× 3), 21.3, 18.4, 13.7, 12.9, 1.1, −5.3 ppm. HRFABMS: calcd for C_23_H_42_O_5_NaSi [M+Na]^+^ 426.2802, found: 426.2804.

*Synthesis of (E)-4-acetoxy-5-(3-(((tert-butyldimethylsilyl)oxy)methyl)-3-methyloxiran-2-yl)-3-methyl- pent-2-en-1-yl pivalate* (**8**, mixture of diastereoisomers). 3-Chloroperoxybenzoic acid (348.65 mg, 2.02 mmol) in anhydrous CH_2_Cl_2_ (19 mL) was added to a solution of acetylated derivate of **7** (431 mg, 1.01 mmol) in anhydrous CH_2_Cl_2_ (20 mL) under an argon atmosphere at 0 °C, and the mixture was stirred for 2.5 h. Then, CH_2_Cl_2_ (25 mL) was added and the mixture successively washed with saturated aq. NaHCO_3_ solution and brine, dried with anhydrous Na_2_SO_4_, filtered, and evaporated to give a crude residue which yielded 402 mg (0.91 mmol, 90% yield) of **8** after chromatography purification (hexane/*t*-BuOMe, 4:1). ^1^H-NMR (400 MHz, CDCl_3_): δ 5.56 (1H, bt), 5.26 (1H, m), 4.54 (2H, d), 3.50 (2H, s), 2.84 (1H, m), 2.05 (3H, s), 1.85 (2H, m), 1.66 (3H, s), 1.22 (3H, s), 1.16 (9H, s), 0.82 (9H, s) and 0.00 (6H, s) ppm. ^13^C-NMR (100 MHz, CDCl_3_): δ 178.3, 169.9, 169.8, 138.1, 137.8, 122.1, 122.0, 75.7, 75.6, 67.5, 67.4, 60.8. 60.6, 60.4, 57.3, 57.1, 38.7, 32.3, 32.2, 29.7, 27.1 (× 3), 25.8 (× 3), 21.1, 18.3, 12.8, 1.0 and −5.4 ppm. HRFABMS: calcd for C_23_H_42_O_6_NaSi [M+Na]^+^ 465.2648, found: 465.2645.

*Synthesis of ((1R,5S,6R)-6-(((tert-butyldimethylsilyl)oxy)methyl)-5-hydroxy-2,6-dimethylcyclohex-2-en-1-yl)methyl pivalate* (**9**). A mixture of Cp_2_TiCl_2_ (60.5 mg, 0.243 mmol) and Mn dust (356 mg, 6.48 mmol) in strictly deoxygenated THF (5.0 mL) under argon, was stirred at room temperature until the red solution became green. A solution of the corresponding epoxide **8** (360 mg, 0.81 mmol), 2,4,6-collidine (0.74 mL, 5.67 mmol), and TMSCl (0.41 mL, 3.24 mmol) in strictly deoxygenated THF (2.0 mL) was then added, and the mixture was stirred until disappearance of the starting material (6 h) was observed. The reaction was quenched with HCl (2 N, dropwise addition of 10 mL), extracted with *t*-BuOMe (3 × 20 mL), washed with brine, dried with anhydrous Na_2_SO_4_, and concentrated under reduced pressure. Product was purified by flash chromatography on silica gel (hexane/*t*-BuOMe, 7:1) to yield the monocycle **9** (219.2 mg, 0.57 mmol, 70% yield) as a colorless solid. ^1^H-NMR (500 MHz, CDCl_3_): δ 5.39 (1H, bs), 4.30 (1H, dd, *J* = 11.8, 4.4 Hz), 4.07 (1H, dd, *J* = 11.8, 4.3 Hz), 3.85 (1H, dd, *J* = 8.4, 5.6 Hz), 3.68 (1H, d, *J* = 10.0 Hz), 3.53 (1H, d, *J* = 10.0 Hz), 2.22 (2H, m), 2.02 (1H, m), 1.68 (3H, s), 1.19 (9H, s), 0.89 (9H, s), 0.88 (3H, s), 0.07 (3H, s) and 0.06 (3H, s) ppm. ^13^C-NMR (125 MHz, CDCl_3_): δ 178.5, 132.6, 121.1, 71.5, 68.9, 62.7, 43.1, 41.4, 38.8, 30.8, 27.2, 25.9, 22.0, 18.2, 12.0 and −5.5 ppm. HRFABMS: calcd for C_21_H_40_O_4_SiNa [M+Na]^+^ 407.2594, found: 407.2593.

## 4. Conclusions

As a conclusion, the stereoselective synthesis of a highly functionalized cyclohexane key intermediate has been accomplished. This enables potential access to both the labdane and trisporoid families. The main objective is to facilitate the approach to the widest structural diversity of these compounds for their use as a standard either in subsequent identifications, quantifications, and biological activity tests. The final and most important step of the sequence consists of a domino process including homolytic epoxide opening, Ti(III)-mediated stereoselective catalytic cyclization, and regioselective elimination of an –OAc group of a conveniently functionalized epoxypolyprene. The proper functionalization of the oxiranic intermediate allows one to obtain the final product with complete stereocontrol in up to five of the six carbons of the final cyclohexane structure.

## Supplementary Materials

Supplementary materials can be accessed at: http://www.mdpi.com/1420-3049/19/2/1748/s1.
