# Supplementary material for: Easy Access to a Cyclic Key Intermediate for the Synthesis of Trisporic Acids and Related Compounds
Source: Molecules. 2014 Feb 3;19(2):1748–62. doi: 10.3390/molecules19021748 (PMC6271007; doi:10.3390/molecules19021748)

Supporting Information

^{1}H- and ^{13}C-NMR spectra for Compounds 4−9

Compound **4** S2

Compound **5** S3

Compound **6** S4

Compound **7** S5

Compound **8** S6

Compound **9** S7

NOE Experiments for **9** S8

Figure S1. ^{1}H and ^{13}C spectra for **4**.

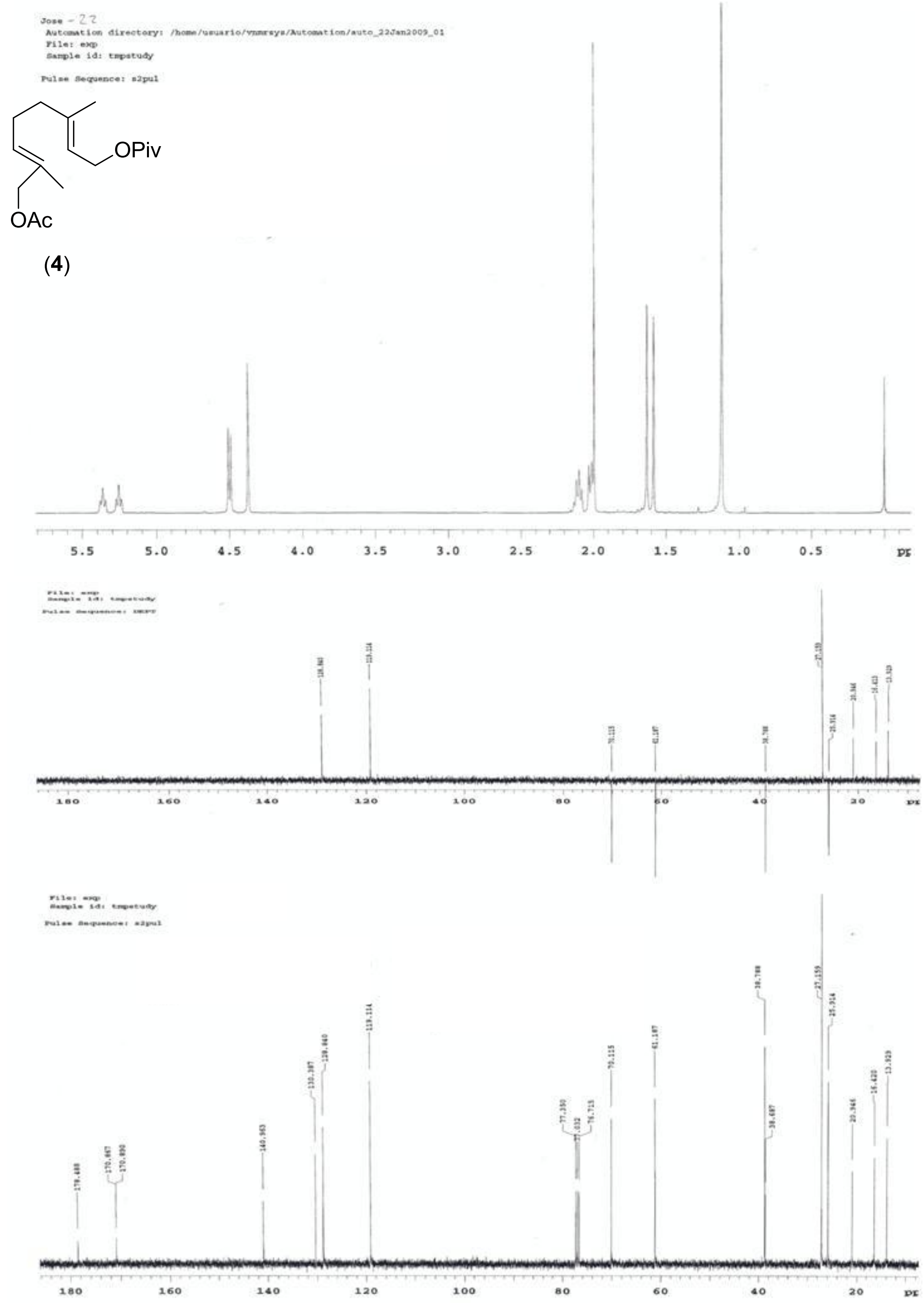

Figure S2. ^{1}H and ^{13}C spectra for **5**.

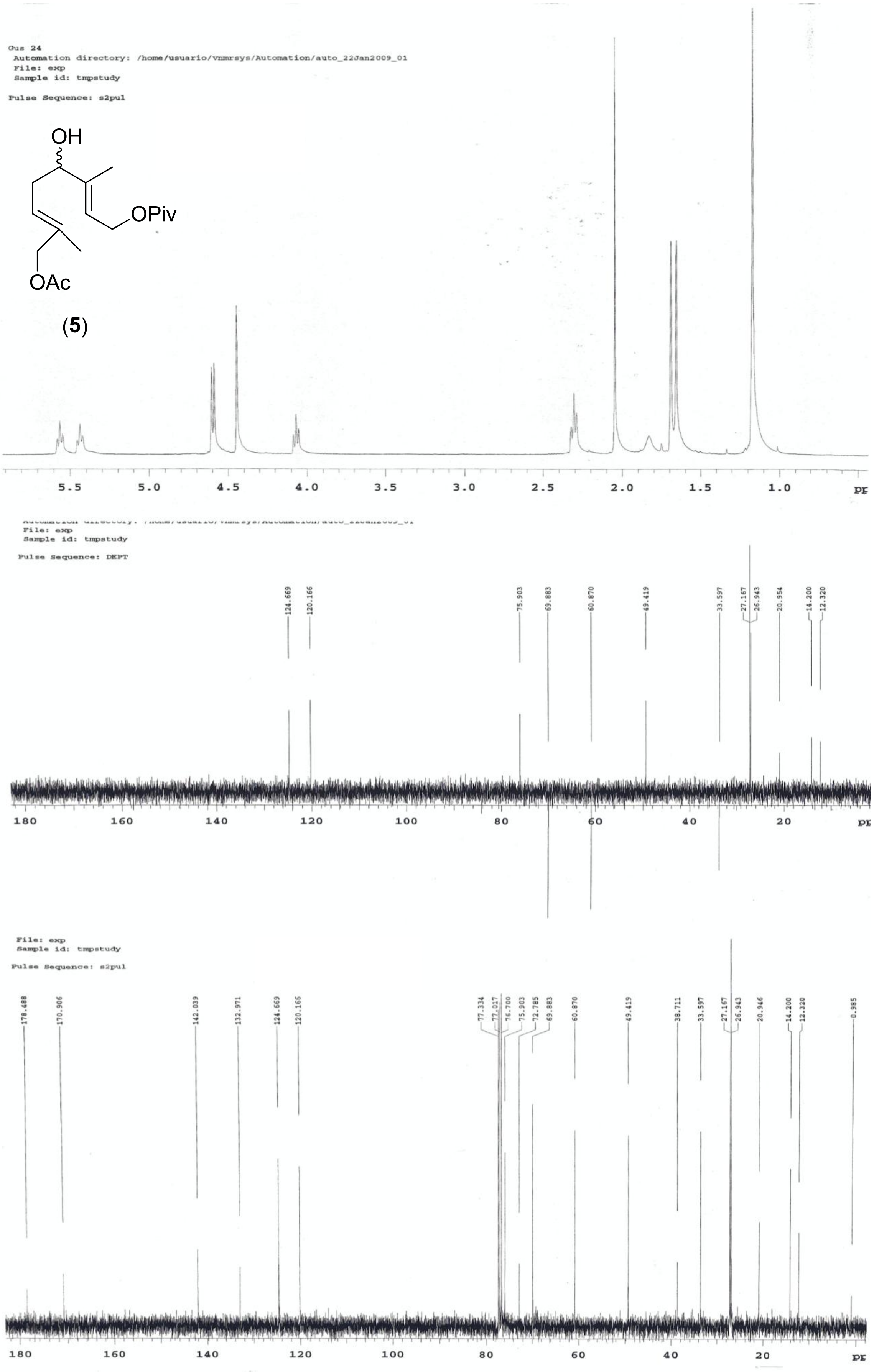

Figure S3. ^{1}H and ^{13}C spectra for **6**.

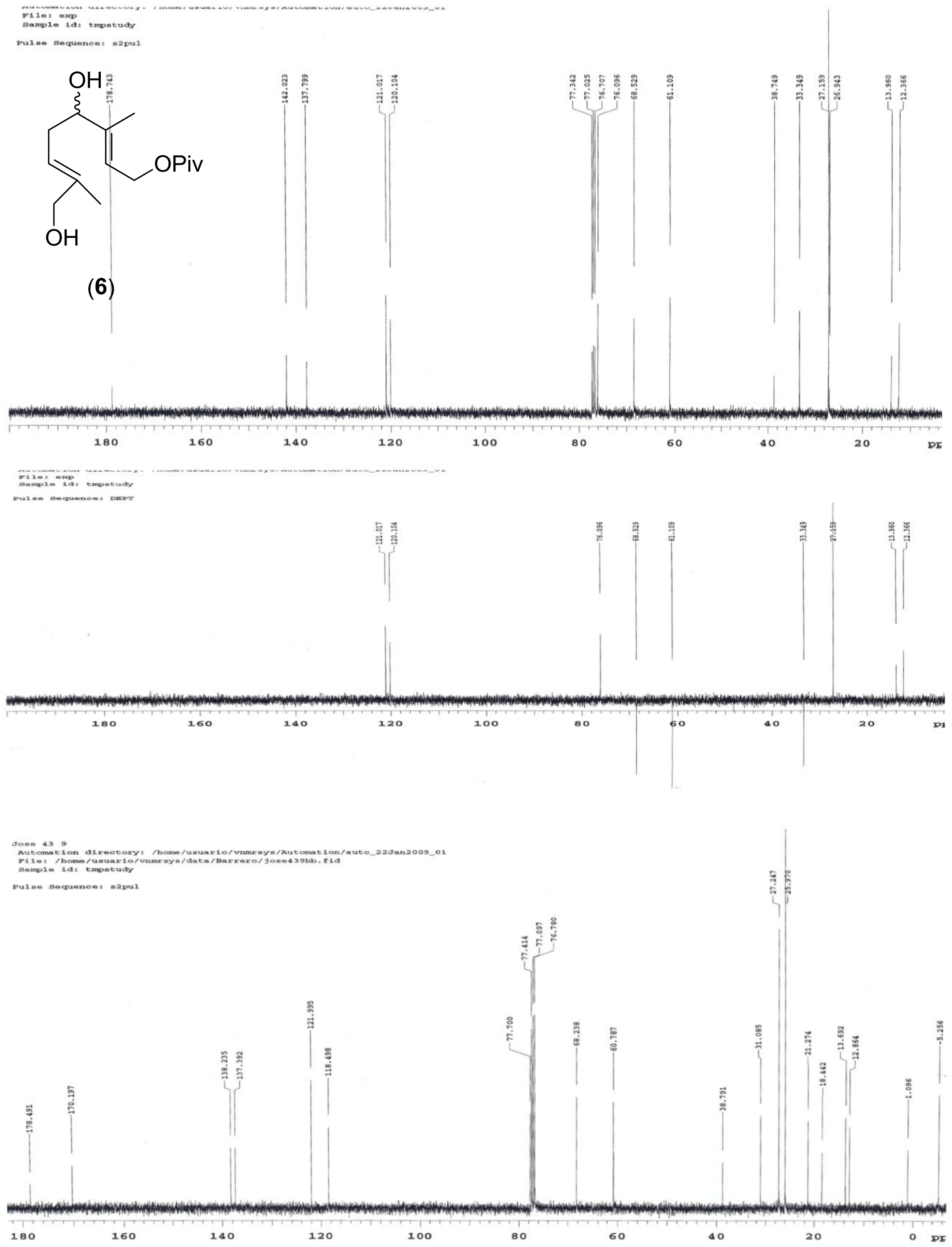

Figure S4. ^{1}H and ^{13}C spectra for **7**.

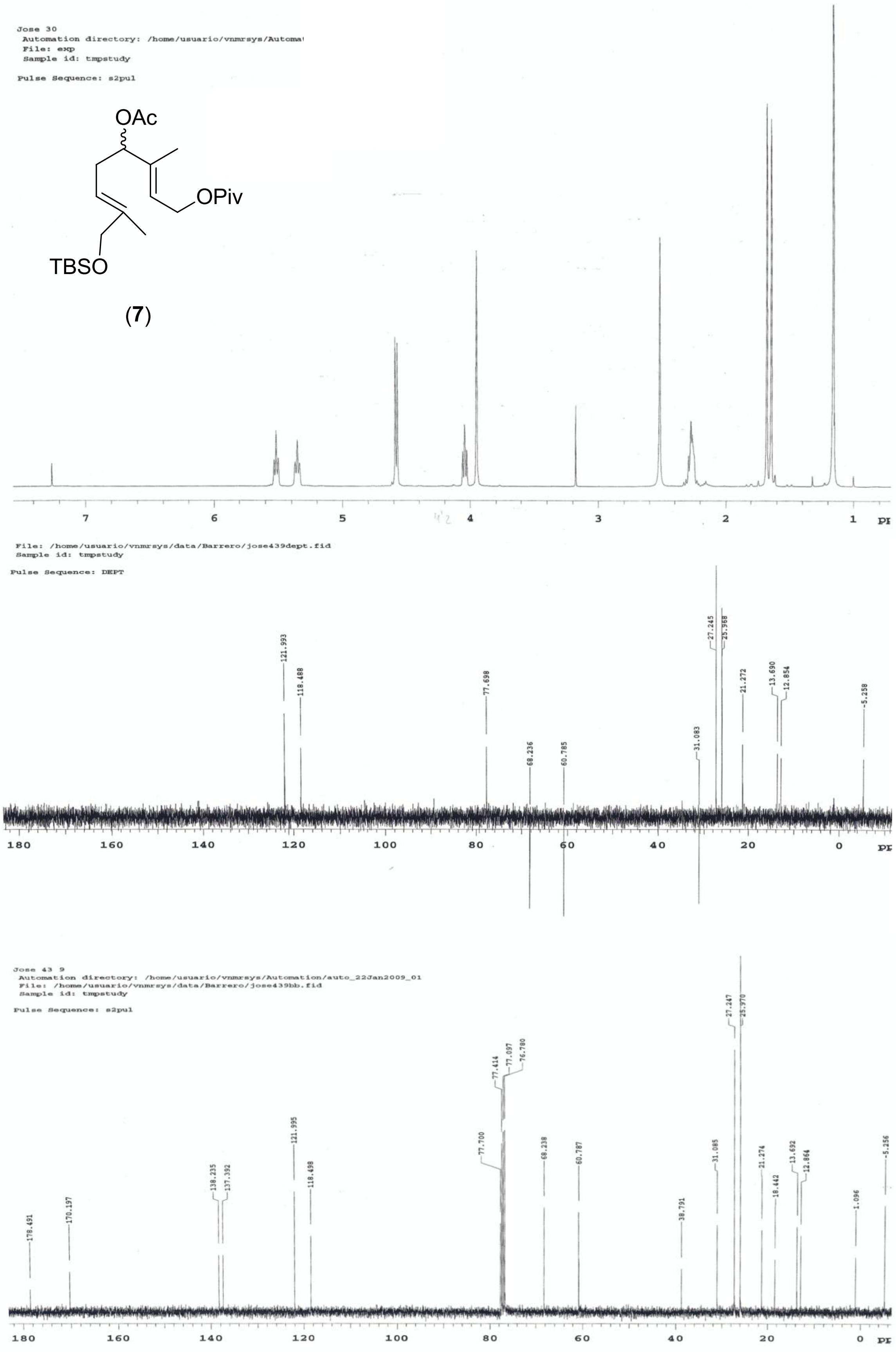

Figure S5. ^{1}H and ^{13}C spectra for **8**.

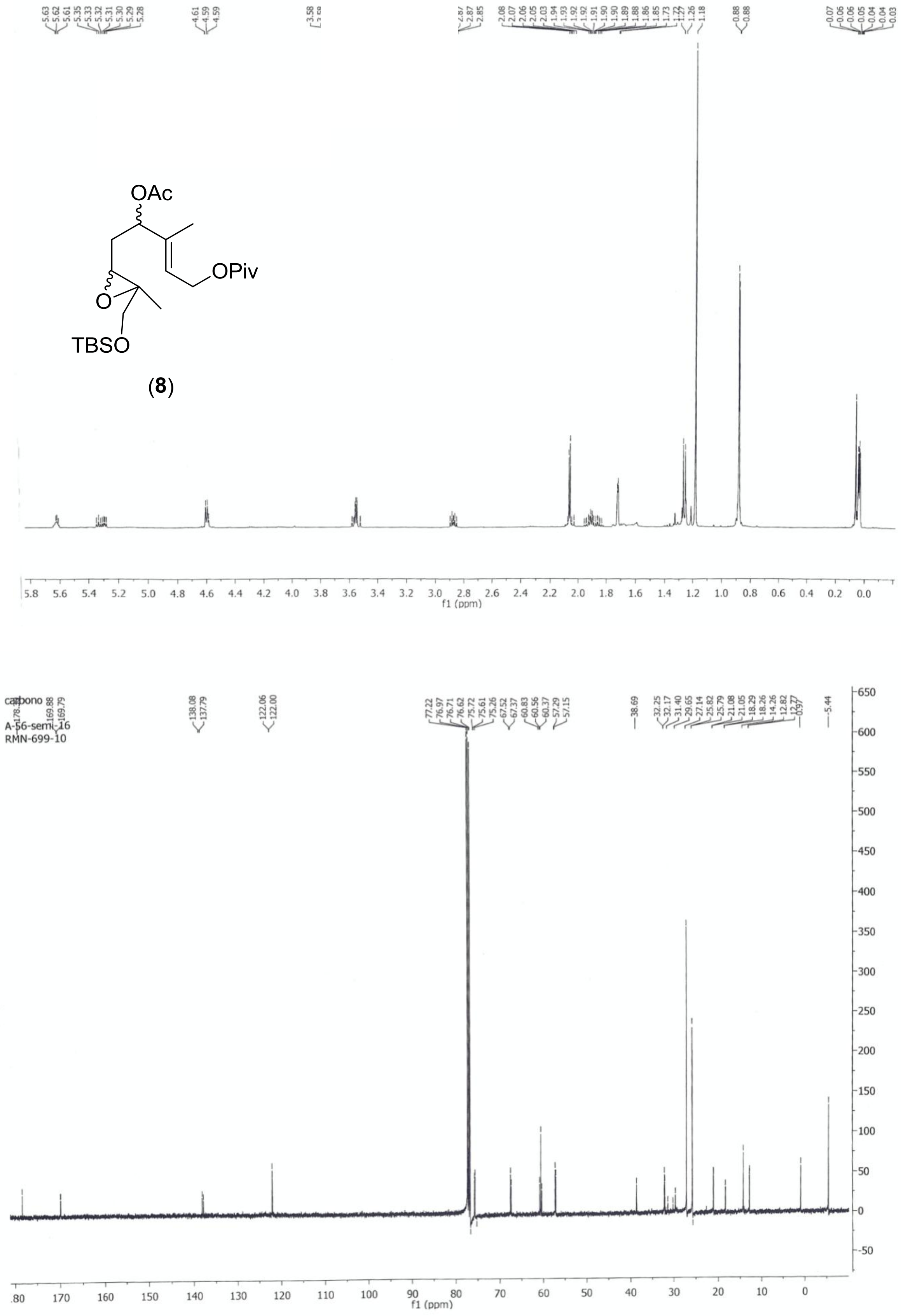

Figure S6. ^{1}H and ^{13}C spectra for **9**.

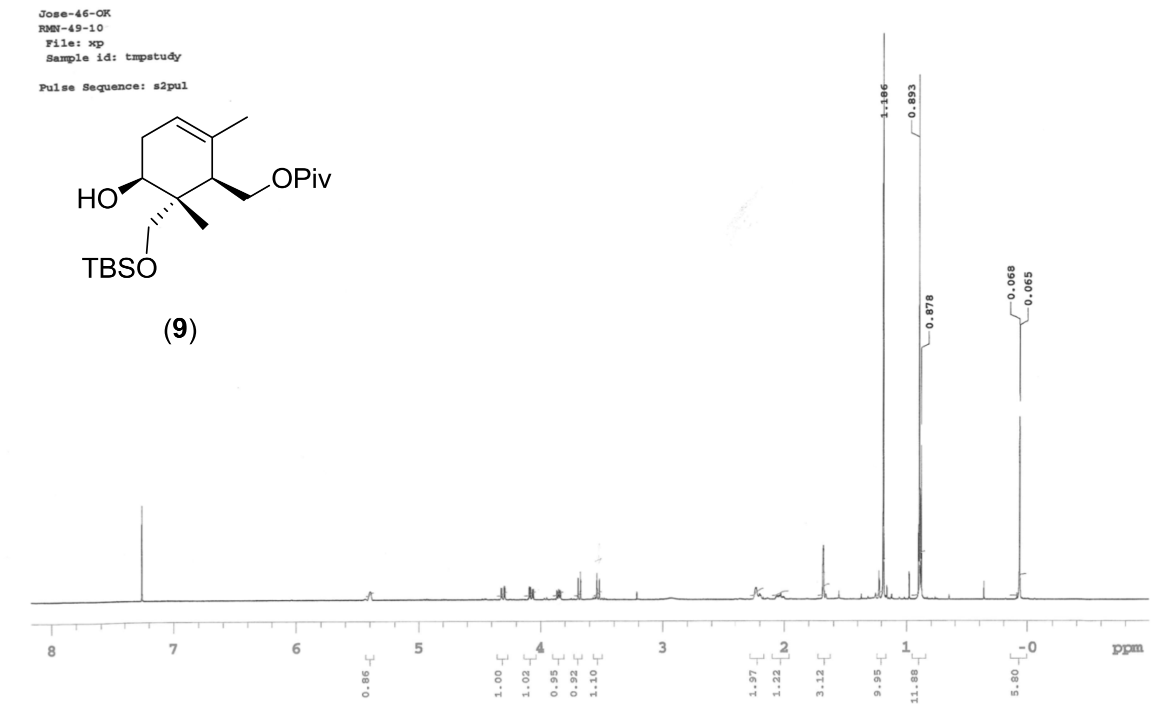

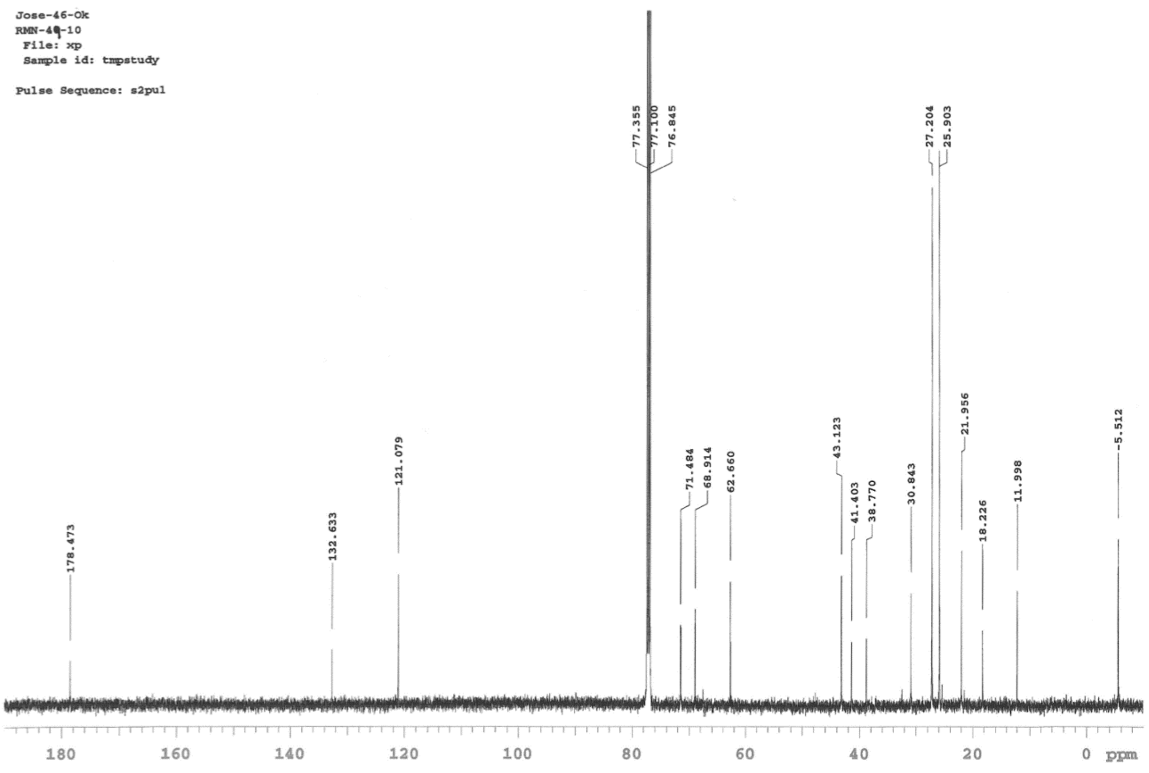

Figure S6. *Cont.*

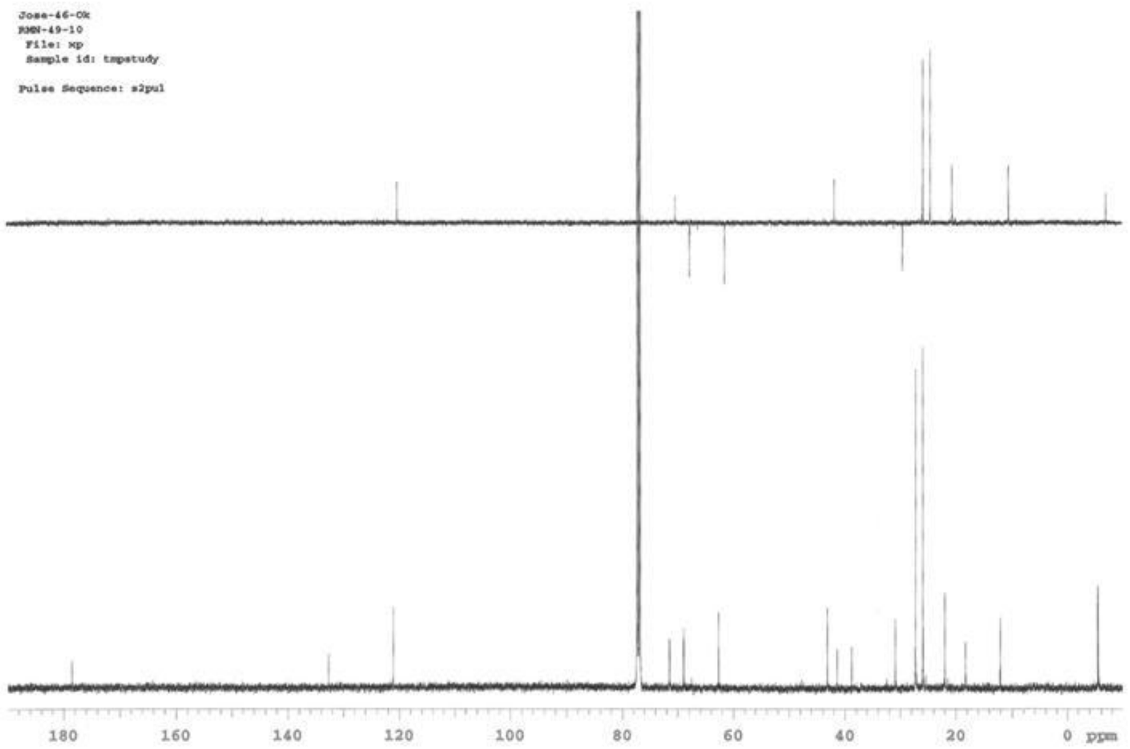

<u>NOE Experiments for **9**</u>

Figure S7. Representative NOE experiment for **9**.

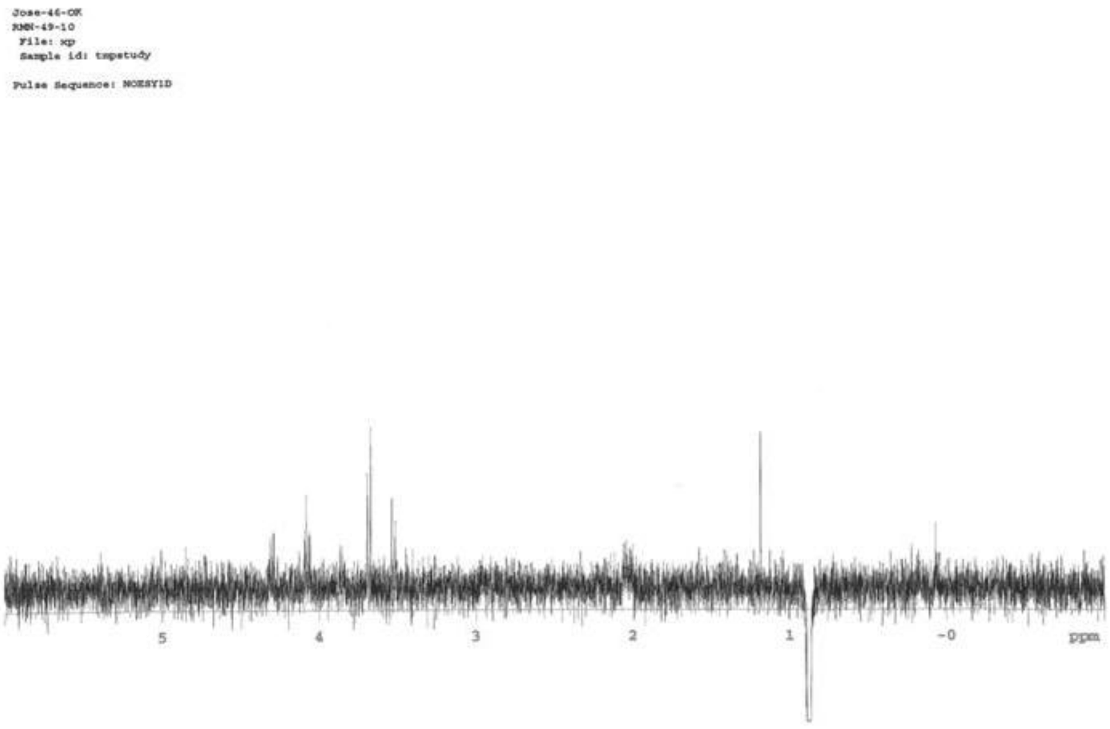

Figure S7. *Cont.*

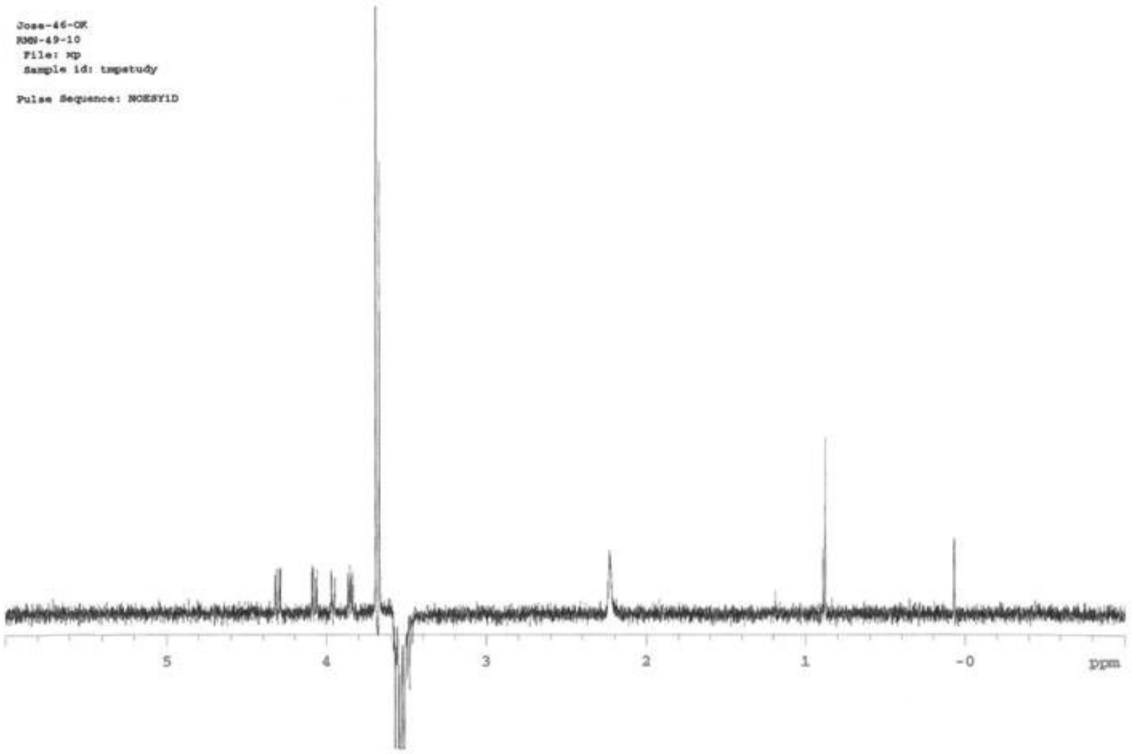

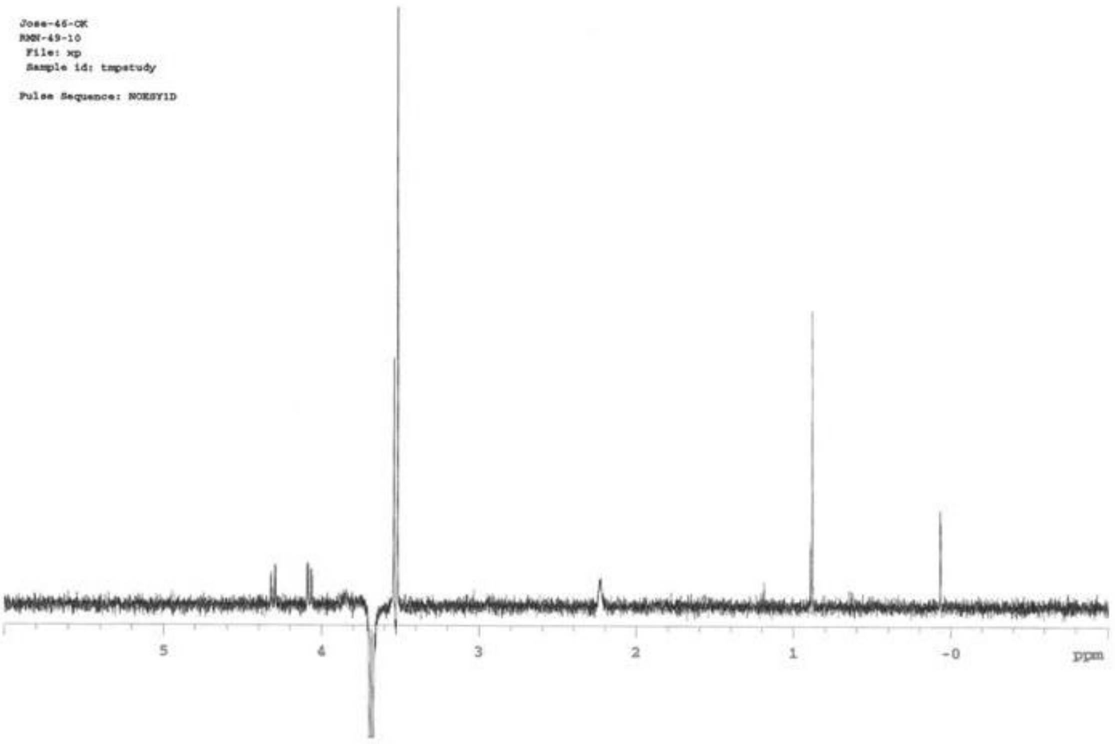

Figure S7. *Cont*.

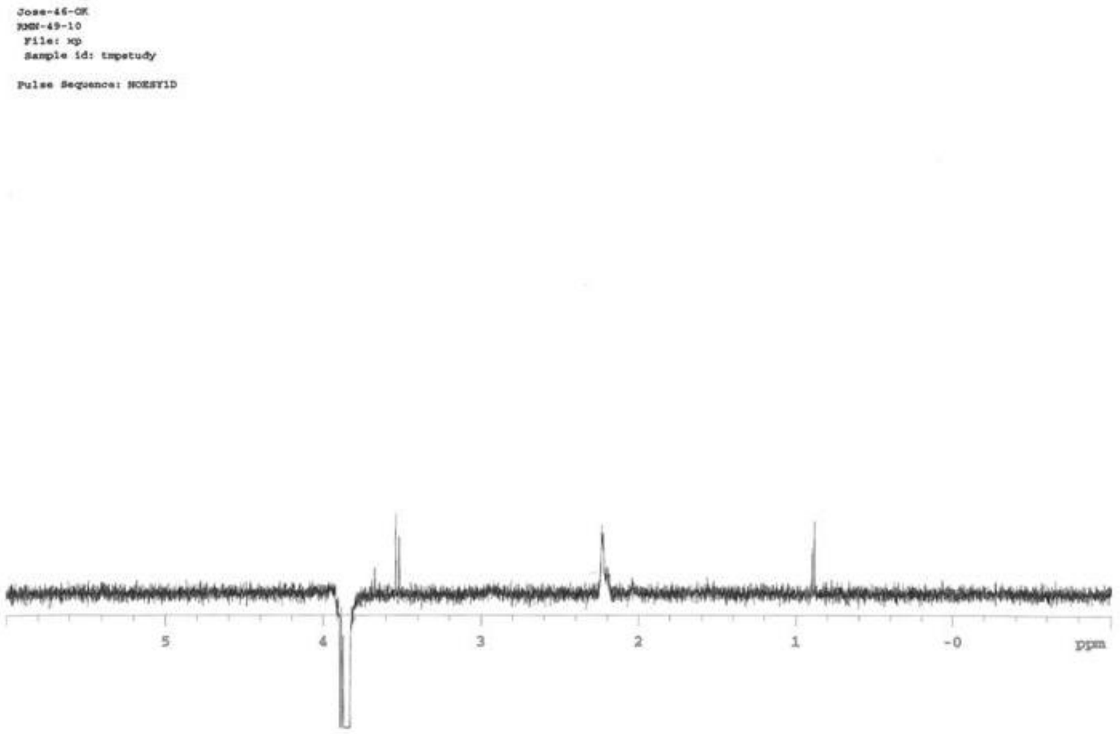

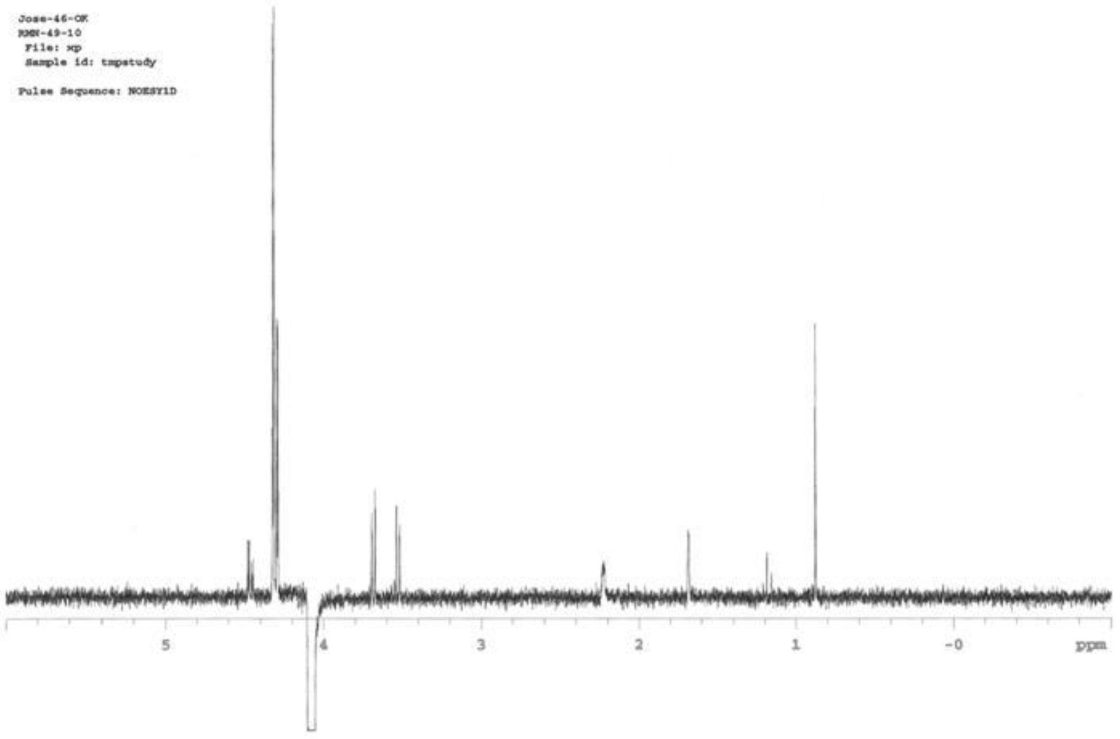

Supplement: Supplementary file 1 [file molecules-19-01748-s001.pdf]
